# Genome-Wide Differential DNA Methylation in Reproductive, Morphological, and Visual System Differences Between Queen Bee and Worker Bee (*Apis mellifera*)

**DOI:** 10.3389/fgene.2020.00770

**Published:** 2020-08-07

**Authors:** Hongfang Wang, Zhenguo Liu, Ying Wang, Lanting Ma, Weixing Zhang, Baohua Xu

**Affiliations:** Laboratory of Nutrition and Physiology of Honeybees, College of Animal Science and Technology, Shandong Agricultural University, Tai’an, China

**Keywords:** *Apis mellifera*, queen bee, worker bee, methylation, reproduction, morphology, vision

## Abstract

There are many differences in external morphology and internal physiology between the *Apis mellifera* queen bee and worker bee, some of which are relevant to beekeeping production. These include reproductive traits, body size, royal jelly secreting properties, and visual system development, among others. The identification of candidate genes that control the differentiation of these traits is critical for selective honeybee breeding programs. In this study, we compared the genomic methylation of queen bee and worker bee larvae at 3, 4, and 5 days of age by whole-genome bisulfite sequencing, and found that the basic characteristics of genomic methylation in queen and worker larvae were the same. There were approximately 49 million cytosines in the *Apis* larvae genome, of which about 90,000 were methylated. Methylated CpG sites accounted for 99% of the methylated cytosines, and methylation mainly occurred in exons. However, methylation levels of queen and worker larvae showed different trends with age: the methylation level of queen larvae varied with age in an inverted parabola, while the corresponding trend for worker larvae with resembled an exponential curve with a platform. The methylation level of queen larvae was higher than that of worker larvae at 3 days of age, lower than that of worker larvae at 4 days of age, and similar to that of worker larvae at 5 days old. The top 10 differentially methylated genes (DMGs) and 13 caste-specific methylated genes were listed, and correlations with caste determination were speculated. We additionally screened 38 DMGs between queen larvae and worker larvae involved in specific organ differentiation as well as reproduction, morphology, and vision differentiation during caste determination. These genes are potential molecular markers for selective breeding of *A. mellifera* to improve fecundity, royal jelly production, body size, and foraging, and represent candidate genes for investigating specialized functional segregation during the process of caste differentiation.

## Introduction

Honeybees are social insects which possess the ability to develop into two castes of organisms with different morphological and physiological features, with the same genome, in response to environmental cues (Winston, 1991; Groh and Rössler, 2008). This fascinating biological phenomenon is referred to as caste differentiation. There are many differences in characteristics between worker bees and queen bees. Here, we focus on traits relevant to beekeeping production. For instance, the queen is larger than the worker bee (Streinzer et al., 2013; Wang et al., 2015; He et al., 2017) and fertile, while the worker is sterile (Michener, 1974; Wilson, 1975; Koeniger et al., 2011). Worker bees have superior eyesight to queen bees, which is useful for foraging (Ribi et al., 1989; Streinzer et al., 2013) and, unlike queens, can secrete royal jelly (Drapeau et al., 2006). The selection of candidate genes related to these traits should establish a strong theoretical foundation for bee selective breeding and beekeeping.

Nutrition is a key environmental cue that induces caste differentiation (Haydak, 1943; Weaver, 1955; Kucharski et al., 2008; Maleszka, 2014). Many nutrients are different between royal jelly and worker jelly (Wang et al., 2016). Furthermore, the alternative developmental pathways of queen and worker bees are also associated with subtle transcriptional changes (Barchuk et al., 2007). More than 4,500 differentially expressed genes (DEGs) between queen and worker in the larval stage have been reported (Chen et al., 2012), supporting the tenet that differential expression of genes controls caste differentiation. Therefore, it is generally accepted that while nutrition is the external cue for caste differentiation, differential gene expression is the internal mechanism involved. How do honeybees integrate external nutritional signals into internal genetic signals? As reported by Elango et al. (2009), DNA methylation regulates gene expression during honeybee caste determination. Epigenetic mechanisms are considered the bridge linking the genes to the environment (Maleszka, 2014). In 2008, a study found that nutritional cues that would have normally induced development into the worker resulted in queens or queen-like adults if Dnmt3 was knocked down (Kucharski et al., 2008). This result established the important role of DNA methylation in caste differentiation in the honeybee. The comparison of methylome between queen and worker using bee brain tissue (Lyko et al., 2010), larval head (Foret et al., 2012), and larvae (Shi et al., 2013) revealed bee methylation features and the mechanisms (alternative splicing) by which DNA methylation regulates gene expression (Flores et al., 2012). However, none of these studies focused on candidate genes associated with the differences in traits between queen and worker. The purpose of our study is to identify these candidate genes, using genome-wide methylation sequencing, to provide a foundation for investigation of the specific role of these candidate genes in tissue and organ differentiation between honeybee castes.

## Materials and Methods

### Honeybee Larvae Sampling and DNA Preparation

Larvae (45 queens and 90 workers) were collected from three colonies headed by sister queens artificially inseminated with semen from the same single drone and frozen on dry ice immediately (15 queens and 30 workers/hive). The sampling times were at 3, 4, and 5 days post hatching from eggs (5 queens and 10 workers/day). The detailed sampling method is referred to the reports of Li et al. (2010) and Begna et al. (2011, 2012). DNA was Isolated using the Universal Genomic DNA Extraction Kit (TaKaRa, DV811A). The purity of DNA was evaluated using a K5500 spectrophotometer to ensure that the A260/A280 ratio of DNA was in the range of 1.8 ∼ 2.0. Purified DNA (>2 μg/sample) was sent to Annoroad Gene technology Co. Ltd. (Beijing, China) for the whole genomic bisulfite sequencing (WGBS) with a Illumina Hiseq X10 sequencer. Each DNA sample used for sequencing is a mixing pool of the three colonies.

### WGBS and Data Analysis

Genomic DNA extracted from honeybee larvae was used to prepare the WGBS library. The protocol for library building, reads mapping and data analysis referred to the methods of Li et al. (2017). Briefly, DNA was fragmented to 200–300 bp using a Bioruptor. The DNA fragments were end-repaired by filling in or chewing back 3′ and 5′ overhangs, added A-tailing to the repaired 3′ end, Ligated Methylated Adapter, and treated with sodium bisulfite using DNA bisulfite convert kit (TIANGEN, Beijing) and then amplifying by PCR. After quality control of the qualified library, sequencing was performed using an Illumina HiSeq 2500 with PE125 sequencing strategy.

Sequencing data were firstly filtered to remove low-quality reads and obtain usable data. The clean data were aligned to the honeybee reference genome V4.5. Both the *Apis mellifera* genome sequences and the clean reads were transformed into bisulfite-converted version and indexed using Bowtie2 software. The detailed strategy for reads mapping refers to Li et al. (2017).

Differentially methylated regions (DMRs) were identified based on the methylation information of each site using the DSS software (Feng et al., 2014; Wu et al., 2015; Park and Wu, 2016). The core of DSS is a new dispersion shrinkage method for estimating the dispersion parameter from Gamma-Poisson or Beta-Binomial distributions. DSS possess three characteristics to detect DMRs. First, spatial correlation. Proper utilization of the information from neighboring Cytosine sites can help improve estimation of methylation levels at each Cytosine site, and hence improve DMR detection. Second, the read depth of the Cytosine sites provides information on precision that can be exploited to improve statistical tests for DMR detection. Finally, the variance among biological replicates provides information necessary for a valid statistical test to detect DMRs, when there is no biological replicate, DSS combining data from nearby Cytosine sites and using them as ‘pseudo-replicates’ to estimate biological variance at specific locations. According to the distribution of DMRs through the genome, we defined the genes related to DMRs as genes whose gene body region (from TSS to TES) or promoter region (upstream 2 kb from the TSS) have a overlap with the DMRs.

Gene Ontology (GO) enrichment analysis of genes related to DMRs was implemented by the GOseq R package (Young et al., 2010), in which gene length bias was corrected. GO terms with corrected *P*-value less than 0.05 were considered significantly enriched by DMR-related genes. Pathway annotation information was obtained from the Kyoto Encyclopedia of Genes and Genomes (KEGG) website^1^. We used KOBAS software (Mao et al., 2005) to test the statistical enrichment of DMR related genes in KEGG pathways.

## Results

### Data Quality of Whole-Genome Methylation Sequencing

The sequencing of bisulfite-converted honeybee DNA yielded six datasets of 391 million reads after filtration and quality checks (Table 1). Of these, 75.2% were mapped to unique genomic regions (Table 2). The total sequence output was 48.9 Gb (8.0 Gb for the 3d-queen larvae, 8.2 Gb for the 4d-queen larvae, 8.1 Gb for the 5d-queen larvae, 8.1 Gb for the 3d-worker larvae, 8.2 Gb for the 4d-worker larvae, and 8.3 Gb for the 5d-worker larvae) yielding a combined 33× coverage of the 250 Mb genome (Table 1). The average sequencing error rate was 0.46% (Table 2).

**TABLE 1:** The dataset quality and quantity of whole genome methylation sequencing.

Sample	Q3d	Q4d	Q5d	W3d	W4d	W5d
Reads length (bp)	125	125	125	125	125	125
Raw reads	78,792,046	83,673,606	78,765,054	82,555,882	79,376,440	81,455,292
Raw bases (bp)	9,849,005,750	10,459,200,750	9,845,631,750	10,319,485,250	9,922,055,000	10,181,911,500
Clean reads	63,997,178	65,824,196	64,560,700	64,641,498	65,520,538	66,648,338
Clean reads rate (%)	81.223	78.668	81.966	78.3	82.544	81.822
Clean bases (bp)	7,999,647,250	8,228,024,500	8,070,087,500	8,080,187,250	8,190,067,250	8,331,042,250
Low-quality reads	13,372,102	12,624,708	13,251,950	12,860,288	12,757,290	12,841,852
Low-quality reads rate (%)	16.971	15.088	16.825	15.578	16.072	15.766
Depth (×)	32	33	32	32	33	33
Ns reads	8,130	8,812	9,338	8,588	8,532	9,784
Ns reads rate (%)	0.01	0.011	0.012	0.01	0.011	0.012
Adapter polluted reads	1,414,636	5,215,890	943,066	5,045,508	1,090,080	1,955,318
Adapter polluted reads rate (%)	1.795	6.234	1.197	6.112	1.373	2.4
Original Q30 bases rate (%)	94	94.422	94.035	94.189	94.265	94.291
Clean Q30 bases rate (%)	98.364	98.496	98.289	98.416	98.371	98.386

**Ns read, the sequence concluding excessive N bases*.*

**TABLE 2:** The data mapping of WGMS.

Sample	Q3d	Q4d	Q5d	W3d	W4d	W5d
Clean reads	63997178	65824196	64560700	64641498	65520538	66648338
Mapped reads	49,939,932	52,243,524	52,463,416	51,160,814	53,026,264	53,574,794
Mapped ratio (%)	78	79	81	79	81	80
Unique mapped reads	46,841,338	49,372,196	49,692,964	47,648,910	49,867,246	50,698,370
Unique mapped ratio (%)	73.2	75	77	73.7	76.1	76.1
Insert size (bp)	183	169	193	166	182	177
Error rate (%)	0.4259	0.4316	0.4729	0.4074	0.6169	0.417
Duplicated rate (%)	4.93	6.6	6.73	4.3	6.64	5.11

### Methylome Profiles in Queen Larvae and Worker Larvae

**FIGURE 1:**
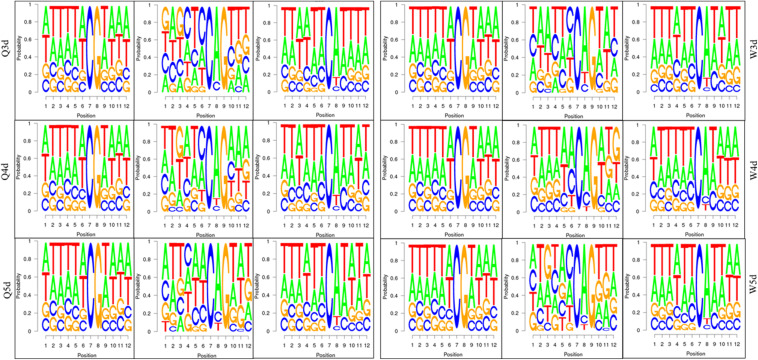
The bases bias around mCG, mCHG, and mCHH.

**FIGURE 2:**
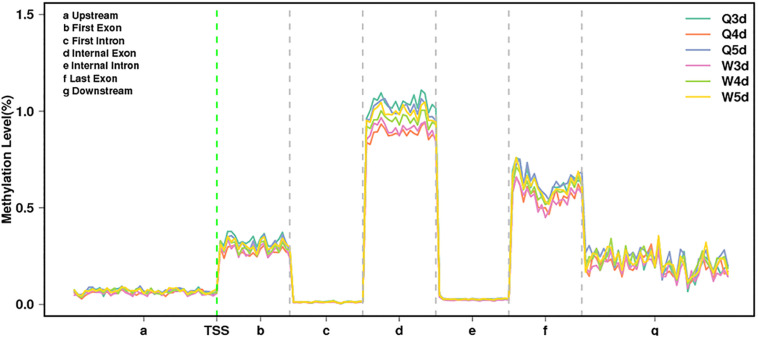
The methylation levels of queen larvae and worker bee larvae at 3, 4, and 5 days old in different transcription elements. TSS, transcriptional start site.

As shown in Table 3, the *A. mellifera* larvae genome comprises approximately 49 million cytosines; among these, only about 90,000 cytosines were found to be methylated. The cytosines in the genome of the *A. mellifera* larvae were classified into three types, namely CG, CHG, and CHH; of these, the most numerous were found to be CHH (about 29 million); the most rare, CHG (about 6 million); and the number of CG was 14 million. Nearly all the methylated cytosines occurred in CpG dinucleotides. The methylated CpG (mCG) account for 99% of the methylated cytosines (mC). The bases surrounding mCG, mCHG, and mCHH possessed bias. A and T highly probability appeared before and behind mCG and mCH (Figure 1). In the *A. mellifera* genome, the majority of this methylation occurred in exons. The methylation level in internal exons was the highest, followed by the last exon, first exon, and downstream; in contrast, the methylation level of upstream and introns were lower (Figure 2).

**TABLE 3:** The number and proportion of mC, mCG, mCHG, and mCHH.in queen larvae and worker larvae.

Sample	Q3d	Q4d	Q5d	W3d	W4d	W5d	Average
C	50,099,650	47,898,135	49,747,594	49,336,141	48,314,824	49,674,421	49,178,461
CG	14,155,587	13,430,217	13,789,286	13,980,714	13,350,575	13,762,330	13,744,785
CHG	6,219,457	5,942,180	6,123,791	6,141,106	5,948,607	6,120,850	6,082,665
CHH	29,724,606	28,525,738	29,834,517	29,214,321	29,015,642	29,791,241	29,351,011
mC	97,367	85,173	102,746	83,608	94,568	100,652	94,019
mCG	96,747	84,603	102,033	83,158	93,967	100,058	93,428
mCHG	76	72	87	66	92	83	79
mCHH	544	498	626	384	509	511	512
mCG/mC (%)	99.36	99.33	99.31	99.46	99.36	99.41	99.37
mCHG/mC (%)	0.08	0.08	0.08	0.08	0.1	0.08	0.08
mCHH/mC (%)	0.56	0.58	0.61	0.46	0.54	0.51	0.54

**FIGURE 3:**
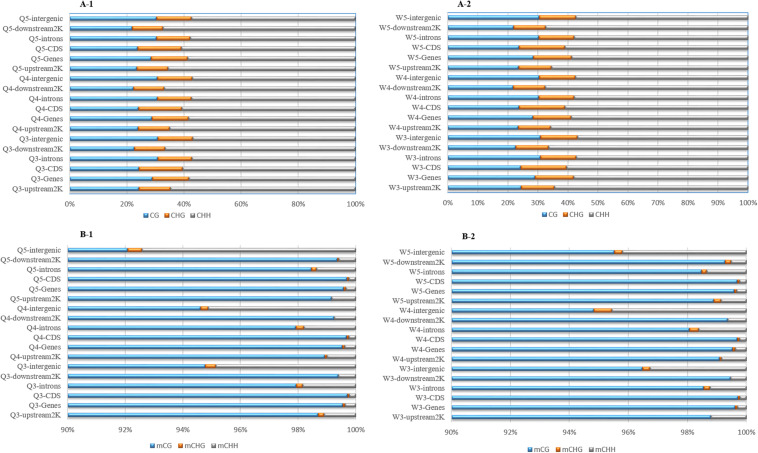
The location and proportion of various C site types. **(A-1)** The location and proportion of CG, CHG, and CHH in queen larvae at 3, 4, and 5 days old. **(A-2)** The location and proportion of CG, CHG, and CHH in worker larvae at 3, 4, and 5 days old. **(B-1)** The location and proportion of mCG, mCHG, and mCHH in queen larvae at 3, 4, and 5 days old. **(B-2)** The location and proportion of mCG, mCHG, and mCHH in worker larvae at 3, 4, and 5 days old.

**FIGURE 4:**
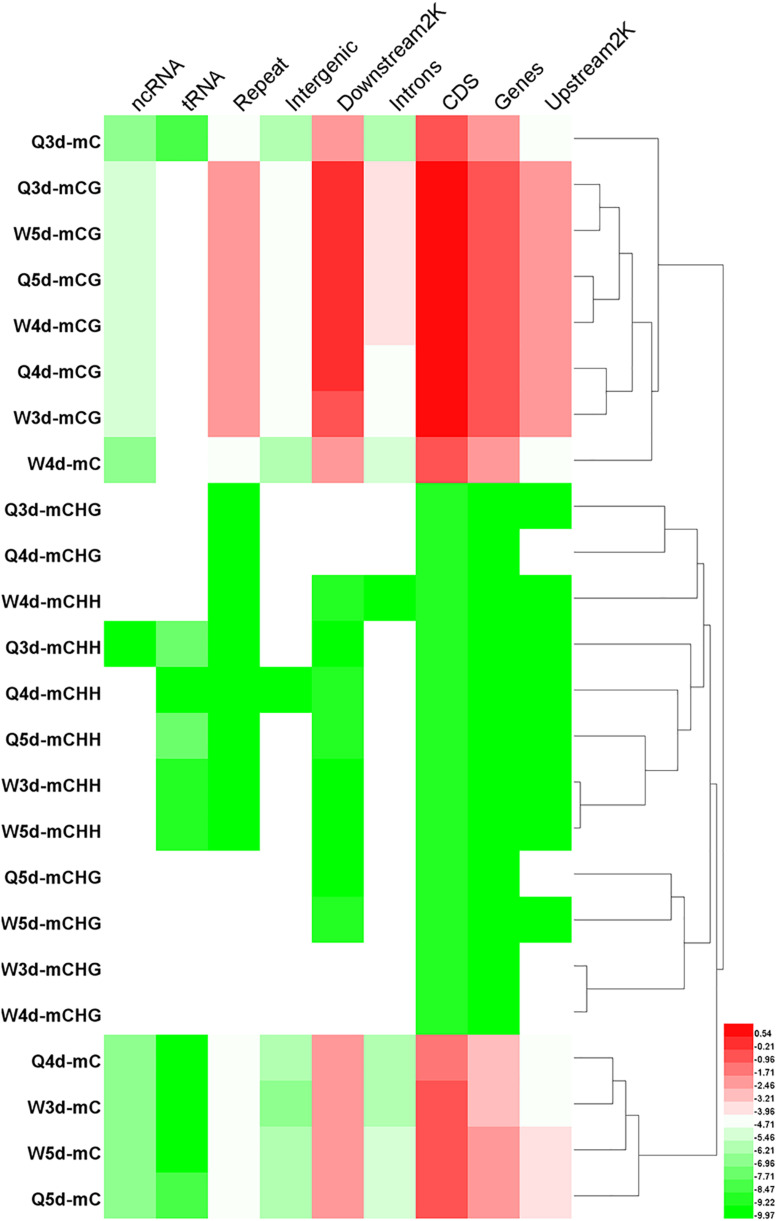
The methylation levels of mC, mCG, mCHG, and mCHH in various genomic location. The methylation levels of the single C site = 100 × The numbers of the reads supporting the methylated C site/The numbers of the reads supporting the methylated C site + The numbers of the reads supporting the un-methylated C site. Regional methylation level at C site = 100 × The sum of methylation levels of all C site in this region/The numbers of C sites, whose depth was not less than 5. Regional methylation level at CG site = 100 × The sum of methylation levels of all CG site in this region/The numbers of CG sites, whose depth was not less than 5.

The numbers of methylated C (Figure 3) and the methylation levels (Figure 4) in different functional genomic regions were analyzed. As shown in Figure 3, the location and proportion of C in the queen and worker larval genomes were the same (Figures 3A-1,A-2). However, the location and proportion of mC varied with caste and age (Figures 3B-1,B-2). Regardless of caste and age, the proportion of mCpG in the CDS region was higher than in other regions. By contrast, the intergenic region exhibited the lowest proportion of mCpG, but the highest proportion of methylated non-CpG. The DNA methylation levels of nine different genomic regions, including 2 Kb upstream, genes, CDS, introns, 2 Kb downstream, intergenic regions, repeats, tRNA, and ncRNA, were investigated using heat map clustering analysis (Figure 4). Generally, the dominant methylation pattern in the *Apis* genome was mCpG. In the CpG context, the CDS region showed a higher methylation level than the other genomic regions, followed by the 2 Kb downstream region, and then genes, repeats, 2 Kb upstream, introns, intergenic, tRNA, and ncRNA, in that order.

### The Divergence of Methylation Characteristics Between Queen Larvae and Worker Larvae

**FIGURE 5:**
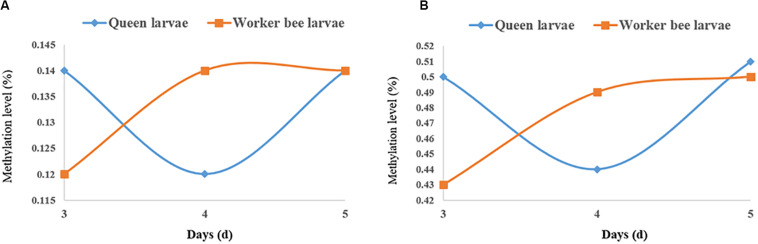
The methylation levels of queen larvae and worker bee larvae at 3, 4, and 5 days old. **(A)** mC level. **(B)** mCpG level. The methylation levels of the single C site = 100 × The numbers of the reads supporting the methylated C site/The numbers of the reads supporting the methylated C site + The numbers of the reads supporting the un-methylated C site. Regional level of methylation (C) = 100 × The sum of methylation levels of all C site in this region/The numbers of C sites, whose depth was not less than 5. Regional level of methylation (CG) = 100 × The sum of methylation levels of all CG site in this region/The numbers of CG sites, whose depth was not less than 5.

The quantities of methylated CpGs (mCpGs) diverged between queen larvae and worker larvae with age; these were 96,747, 84,603, and 1,02,033 mCpG, respectively, for 3, 4, and 5 days queen larvae, and 83,158, 93,967, and 1,00,058 mCpG, respectively, for 3, 4, and 5 days worker larvae (Table 3). The trends in methylation level from 3 to 5 days of age were also different between queen larvae and worker larvae (Figure 5). It was found that the methylation levels of mC and mCpG varied according to similar trends with caste and age. The methylation levels of mC and mCpG in the queen larvae were higher than in the worker larvae at 3 days old. Thereafter, the methylation levels of queen larvae declined at 4 days old; then, at 5 days old, they increased to the same levels as at the age of 3 days. However, the age-dependent regulation of worker larvae methylation levels was completely different from that of the queen larvae. Despite the lower worker larvae methylation levels at 3 days old, a sharp increase was found at 4 days old, and this higher methylation level was maintained until 5 days old. This led to the methylation levels of queen larvae being higher than, lower than, and similar to those of worker larvae at, 3, 4, and 5 days old. These findings imply that the larval stage at 4 days old is a critical period of caste differentiation, and that the divergence of larval methylation between the castes at this period may be the decisive switch that triggers caste differentiation.

The proportion of mC (Figure 3) in various functional genomic regions were also different between queen and worker larvae. A significant difference in mC proportion between queen larvae and worker larvae occurred in the intergenic region. The proportion of mCpG in intergenic regions of worker larvae was higher than in queen larvae, especially at 3 days old and 5 days old (worker, 96.48% mCpG for 3-day-old and 95.52% mCpG for 5-day-old; queen, 94.78% mCpG for 3-day-old and 92.09% mCpG for 5-day-old).

### Differentially Methylated Regions and Differentially Methylated Genes Between Queen Larvae and Worker Larvae

**FIGURE 6:**
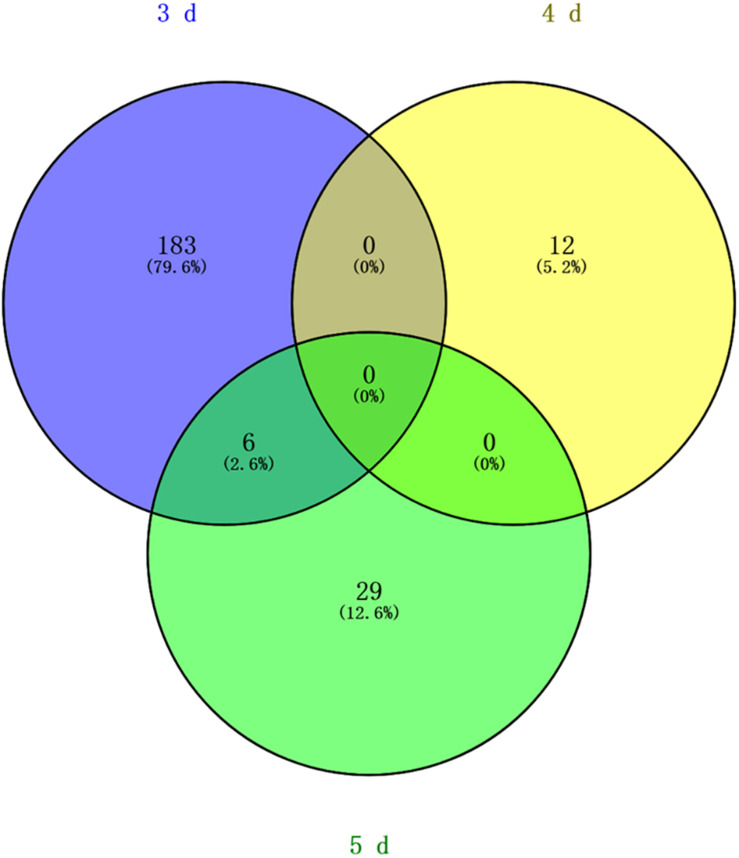
Venn analysis. Blue, different methylated genes between queen larvae and worker larvae at 3 days old; yellow, different methylated genes between queen larvae and worker larvae at 4 days old; green, different methylated genes between queen larvae and worker larvae at 5 days old.

Differentially methylated regions (DMR) between the queen larvae and worker larvae were identified using swDMR software with rigorous parameters. The distribution of DMR in genetic regions is shown in Table 4. These DMRs were mainly distributed in CDS, followed by introns, and then the upstream 2 Kb region. Between queen larvae and worker larvae, respectively, 253, 15, and 44 DMRs were identified at the age of 3, 4, and 5 days (Supplementary Tables S1–S3) (*P* < 0.05). In order to explore the relationship between DNA methylation and gene transcription, we annotated these DMRs using the genomic location and the annotation information for the *A. mellifera* genome. At 3, 4, and 5 days of age, respectively, 189, 12, and 35 differentially methylated genes (DMGs) were annotated from 253, 15, and 44 DMRs (Figure 6). Compared with the queen larvae, the worker larvae had 73, 6, and 19 hyper-methylated genes and had 116, 6, and 16 hypo-methylated genes at 3, 4, and 5 days of age, respectively (Supplementary Figure S1). The top 10 DMGs according to methylation level fold change are shown in Table 5. The 30 DMGs in Table 5 were deemed as the focal DMGs for the subsequent GO and KEGG analyses.

**TABLE 4:** The distribution of DMR in genetic regions.

Region	W3d vs. Q3d		W4d vs. Q4d		W5d vs. Q5d	
	Hyper	Hypo	Hyper	Hypo	Hyper	Hypo
Upstream2K	0	7	4	12	6	3
CDS	279	384	522	522	509	465
Introns	53	35	30	43	71	52

**DMR, differential methylated region. Hyper, high methylation; Hypo, low methylation*.*

**TABLE 5:**
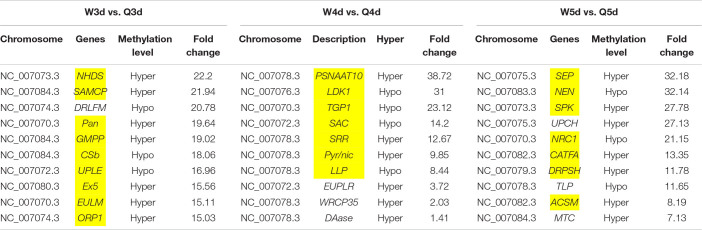
Top 10 different methylated genes according to methylation level fold change between queen larvae and worker larvae at 3, 4, and 5 days old.

**ORP1, oxidation resistance protein 1. EULM, E3 ubiquitin-protein ligase MYCBP2. Ex5, exportin-5. UPLE, ubiquitin-protein ligase E3B. CSβ, coatomer subunit beta. GMPP, cGMP-specific 3′,5′-cyclic phosphodiesterase. Pan, patronin. DRLFM, DNA replication licensing factor Mcm5. SAMCP, S-adenosylmethionine mitochondrial carrier protein. NHDS, NAD-dependent histone deacetylase Sir2. PSNAAT10, putative sodium-coupled neutral amino acid transporter 10. LDK1, LIM domain kinase 1. TGP1, tight junction-associated protein 1. SAC, spectrin alpha chain. SRR, sortilin-related receptor. Pyr/nic, pyrazinamidase/nicotinamidase. LLP, longitudinals lacking protein, isoforms H/M/V. EUPLR, E3 ubiquitin-protein ligase RNF185. WRCP35, WD repeat-containing protein 35. DAase, dopamine N-acetyltransferase. SEP, Sushi, von Willebrand factor type A, EGF and pentraxin domain-containing protein 1. NEN, neogenin. SPK, serine/threonine-protein kinase LATS1. UPCH, uncharacterized protein C4orf29 homolog. NRC1, nuclear receptor corepressor 1. CATFA, cyclic AMP-dependent transcription factor ATF-2. DRPSH, double-stranded RNA-binding protein Staufen homolog 2. TLP, Tonsoku-like protein. ACSM, AP-2 complex subunit mu. MTC, metal transporter CNNM2 W3d vs. Q3d, worker larvae vs. queen larvae at 3 days old. W4d vs. Q4d, worker larvae vs. queen larvae at 4 days old. W5d vs. Q5d, worker larvae vs. queen larvae at 5 days old. Yellow background, caste-specific methylated genes (queen or worker).**

### GO and KEGG Analysis of DMGs

In order to investigate the function and pathways of DMGs, all DMGs were analyzed by GO and KEGG analysis. At 3 days old, 1,516 GO terms were annotated significantly (*P* < 0.05) from the DMGs. Of these, 131, 249, and 1,136 GO terms, respectively, belonged to the categories of cellular component, molecular function, and biological process (Supplementary Table S4). At 4 days old, 1,416 GO terms were annotated (*P* < 0.05). There were 151, 252, and 1,013 GO terms, respectively, in the categories of cellular component, molecular function, and biological process (Supplementary Table S5). At 5 days old, there were 1,525 obvious GO terms (*P* < 0.05). Further, there were 125, 283, and 1,117 terms under the categories of cellular component, molecular function, and biological process, respectively (Supplementary Table S6). The above results suggest that a wide range of genes is involved in the regulation of the biological processes underlying caste differentiation. KEGG analysis results showed that only 43 pathways were significantly enriched (*P* < 0.05) [10 pathways for 3 days old, 8 pathways for 4 days old, and 25 pathways for 5 days old (Supplementary Table S7)].

The 30 DMGs in Table 5 were all annotated by GO (Supplementary Table S8), of which, few DMGs were annotated to be directly associated with caste differentiation. *DRLFM* was annotated under the GO categories of cellular process involved in reproduction. *ORP1* was also annotated to take part in regulation of neuron apoptotic process in the brain; it should be noted that the brain is considered a critical organ in the regulation of caste differentiation (Maleszka, 2008; Lyko et al., 2010; Lockett et al., 2012). Other DMGs were mainly annotated to be involved in behavior, response to chemical and biological stimulus, cell and organ morphogenesis and macromolecule metabolic. These functions are all closely related to castes differentiation.

### DMGs Involved in Reproductive Differentiation Between Queen and Worker Bee

It is well known that the queen bee is fertile, while the worker bee is sterile. The DMGs annotated under GO categories associated with female germ-line cyst formation, oviposition, and related processes are shown in Table 6. For example, Protein hu-li tai shao (*phts*), whose methylation level was higher in worker larvae than queen larvae at both 3 and 5 days old, was annotated to be relevant to female germ-line cyst formation by GO analysis. Histone-lysine *N*-methyltransferase ash1 (*ash1*) may participate in the process of oviposition. Ecdysteroid-regulated gene E74 (*ergE74*) and longitudinals lacking protein (*llp*) are likely to regulate oogenesis and gonad development, respectively. Thyroid receptor-interacting protein 13-like (*trip13l*), RING finger protein 17 (*rfp17*), and serine/threonine-protein kinase tousled-like 1 (*spkt1*) may be involved in gamete generation. In addition, other genes of interest that may regulate the reproductive plasticity of the honeybee are listed in Table 6.

**TABLE 6:**
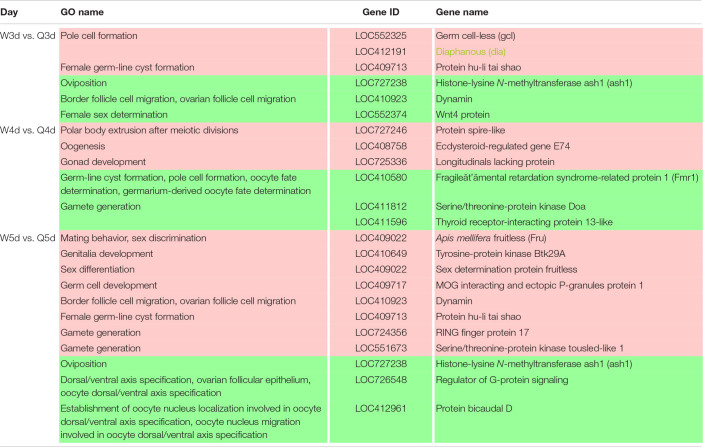
Different methylated genes between queen larvae and worker larvae involved with reproduction by GO annotation.

**Red background, hyper methylated genes worker vs. queen; green background, hypo methylated genes worker vs. queen*.*

### DMGs Involved in Morphological Differentiation Between the Queen and Worker Bee

In addition to the differences in reproductive function, there are additional differences in external morphology between queen and worker bees. For instance, the body size of the queen is larger than that of the worker (Streinzer et al., 2013; Wang et al., 2015; He et al., 2017). The queen’s wings extend to the half of the abdomen or two-thirds of the abdomen, while the worker’s wings almost reach the tail. The worker possesses a pair of appendages of metapedes for collecting pollen, referred to as a pollen basket, while the queen does not. As illustrated in Table 7, some DMGs under the morphogenesis GO background were selected, e.g., *misshapen* (*msn*) was annotated to the GO term of body morphogenesis; *Trithorax group protein osa* (*osa*), *probable Ras GTPase-activating protein* (*rgp*) and *fruitless* (*fru*) were under the GO background of imaginal disk-derived wing/leg morphogenesis. Furthermore, some gland morphogenesis-related GO terms are listed in Table 8. It is believed that the degree of development of the hypopharyngeal gland varies between queen and worker. DMGs including *protein 4.1 homolog* (*p4.1h*), *wnt4 protein* (*wnt4*), *dynamin*, *wnt1 protein* (*wnt1*), *tyrosine-protein kinase Btk29A-like* (*btk29A*), and *crumbs* (*crb*) under the gland morphogenesis GO background are potentially associated with the differential development of hypopharyngeal gland of the female bee.

**TABLE 7:**
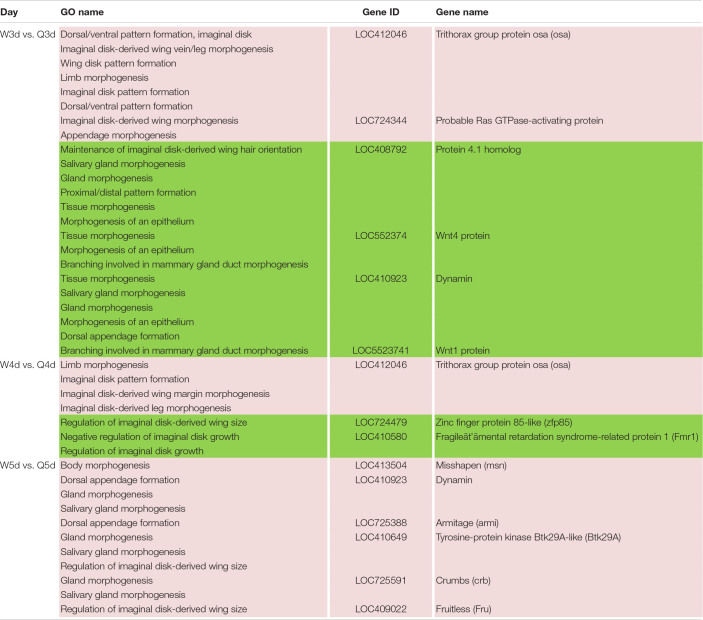
Different methylated genes between queen larvae and worker larvae involved with morphogenesis (leg, wing, gland, and so on) by GO annotation.

**Red background, hyper methylated genes worker vs. queen; green background, hypo methylated genes worker vs. queen*.*

**TABLE 8:**
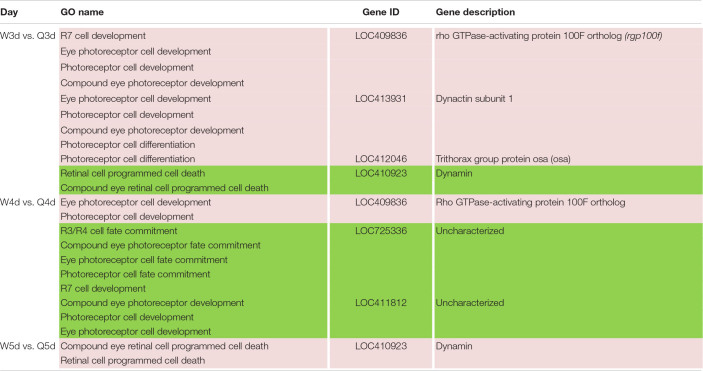
Different methylated genes between queen larvae and worker larvae involved with visual system development by GO annotation.

**Red background, hyper methylated genes worker vs. queen; green background, hypo methylated genes worker vs. queen*.*

### DMGs Involved in Visual System Differentiation Between Queen and Worker Bee

Differences in vision additionally occur between queen and worker. To better adapt to the flying activities outside of nest, the vision of the adult worker bees, whose compound eyes are composed of 3,800–5,900 ommatidia, is superior to that of queen bees whose compound eyes are composed of about 3,500–4,500 ommatidia (Streinzer et al., 2013). As illustrated in Table 8, genes involved in differential development of vision between the two castes are also regulated by DNA methylation. Most of these DMGs are annotated to be related to the development of the eye photoreceptor cell, and include *rho GTPase-activating protein 100F ortholog* (*rgp100f*), *dynactin subunit 1*, *trithorax group protein osa* (*osa*), *LOC725336*, and *LOC411812* (uncharacterized). GO annotation also showed that *dynamin* may regulate compound eye retinal cell programmed cell death. Thus, it was speculated that *dynamin* is an important candidate gene that regulates the number of ommatidia that form the compound eyes.

## Discussion

### Methylome Characteristics of *Apis mellifera*

Caste determination in honeybees is a classic epigenetic phenomenon mediated by nutrition (Haydak, 1943; Weaver, 1955). The study of the honeybee methylome contributes to the understanding of the epigenetic basis of this phenomenon. Previously published studies reveal methylation patterns in the adult brain (Lyko et al., 2010) and larval head (Foret et al., 2012): 70,000 methylated cytosines are reported in the adult brain, and 1,00,000 in the larval head. We found approximately 90,000 methylated cytosines in 3–5 day-old larvae; the majority of this methylation occurred in the exons, and over 99% methylated cytosines occurred in CpG dinucleotides. These findings were consistent with Lyko’s report (2010). However, according to Shi et al. (2013), methylcytosines were most enriched in introns, followed by coding sequence (CDS) regions, in larvae. This results contradict our findings that the methylcytosine (mC/C) rate is 0.78% in CDS, but 0.032% in introns. Most previous reports, however, support our findings that methylation mainly occurs in the exon, which was once thought to be an important distinguishing characteristic of invertebrates and vertebrates (Wang et al., 2006; Suzuki et al., 2007; Zemach et al., 2010). Comparison of the methylomes of *A. mellifera*, *Danio rerio*, and *Mus musculus* also confirm that the methylation level in the exons of *A. mellifera* is higher than in the introns, whereas no such significant trend was observed in *D. rerio* and *M. musculus* (Feng et al., 2010). The level and position of DNA methylation in insects differ from vertebrates. Studies show that many insects genomes display reduced DNA methylation levels compared with vertebrates (Field et al., 2004; Suzuki and Bird, 2008). DNA methylation of vertebrates occurred primarily in promotor region of genes. Promoter methylation tends to inhibit gene transcription (Bird, 1992; Rakyan et al., 2008). But DNA methylation of most insect including honeybees occurred mainly in gene body, so it was proved to be related to alternative splicing (Flores et al., 2012). The location of DNA methylation in insects is species-specific. DNA methylation was found to mainly occur in the introns in *Drosophila melanogaster* (Guan et al., 2019) and *Tribolium castaneum* (Song et al., 2017). Comparison of the methylation of social insects including *Camponotus floridanus* (Bonasio et al., 2012), *Zootermopsis nevadensis* (Glastad et al., 2016), and *Ceratina calcarata* (subsocial insect) (Rehan et al., 2016) revealed that methylation preference for exons was common in social insects. Among these social insect species, *Z. nevadensis* shows the highest evolutionary distance from *A. mellifera* (Misof et al., 2014; Bewick et al., 2017), and its homology with the bee proteome is lower than that with *Drosophila* and *Tribolium* (Terrapon et al., 2014); however, it still exhibits a methylation preference for exons. In *Nasonia vitripennis*, a non-social insect of Hymenoptera (Beeler et al., 2014), and *Bombyx mori* of Lepidoptera (Xiang et al., 2010), methylation also occurs mainly in the exons. The evolutionary time of the jewel wasp is the same as that for *Apis*, and that of the silkworm is longer than that for *Apis*, which was between *Drosophila* and *Tribolium* (Misof et al., 2014; Bewick et al., 2017). Accordingly, it was found that the methylation of social insects always occurred in exons, but the position of methylation in non-social insects was variable.

### Differences in Methylation Between the Two Castes

Comparison of DNA methylation level between queen and worker larvae indicated that the worker larvae showed higher methylation levels at 4-day-old than the queen larvae; in contrast, methylation levels of the queen larvae were higher than those of the worker larvae at 3-day-old, and both levels were similar at 5-day-old. Although this result is somewhat consistent with Shi’s finding that the methylation levels of worker larvae are higher than those of queen larvae at 4-day-old, a difference was found: in the present study, the methylation levels of queen and worker larvae varied dynamically from 3 to 5 days old. In contrast, Shi’s results showed that queen larvae consistently have lower methylation levels than worker larvae from 2 to 6 days old (Shi et al., 2013). Foret et al. (2012) also found that the vast majority of larval (96-h-old) DMGs (1,967, or 82%) were up-methylated in workers, suggesting that queens would have lower levels of methylation than workers. However, a study in adult bees indicated that the methylation levels of mCpG are almost identical in both castes (Lyko et al., 2010). This suggests that the difference in methylation between queen bee and worker bee mainly occur during the larval stage, and little or no difference is seen at the adult stage. Approximately 560 DMGs in the brains of mature adults, and 2,390 DMGs in larval honeybees were found on comparing queens and workers (Lyko et al., 2010; Foret et al., 2012; Herb et al., 2012), providing further evidence that the larval stage is the key period for caste differentiation. Sharply elevated methylation levels of 4-day-old worker larvae also suggest that 4 days old is a critical time point for caste differentiation, and the divergence of the both castes larvae methylation at this period may act as the decisive switch that triggers caste differentiation. The higher juvenile hormone (JH) levels of the queen larvae relative to those of the worker larvae, from 3 days of age to 5 days of age, were deemed an important factor underlying caste differentiation (Wirtz and Beetsma, 1972; Hartfelder and Engels, 1998). The peak JH levels in queen larvae occurred at 4 days old (Hartfelder and Engels, 1998), also supporting the view that this stage (i.e., the age of 4 days) was an important caste differentiation. Many genes, encoding JH receptors genes and all JH-responsive genes, were found to be methylated or differentially methylated between queen larvae and work larvae (Foret et al., 2012). Differential methylation can cause differential expression of the JH-response genes which continue to affect the expressions of more genes (Barchuk et al., 2007). Differential expression of genes caused castes differentiation (Chen et al., 2012). In addition to DNA methylation, histone acetylation is another epigenetic regulation of JH. CREB binding protein (contains histone acetyltransferases domain) and Trichostatin A (histone deacetylases inhibitor) has been shown to affect JH action (Roy and Palli, 2018). These advances supported the significance of epigenetic regulation in caste differentiation.

Studies of other social insects (*P. canadensis, Polistes dominula, Copidosoma koehleri*, and *Melipona scutellaris*) have also shown that methylation frequencies were similar across adult castes (Shaham et al., 2016; Standage et al., 2016; Cardoso-Júnior et al., 2017). Methylation levels in the adult queen of *C. floridanus* were lower than those of the worker; however, no difference between virgin queen and worker was found (Bonasio et al., 2012). In ants, the relative DNA methylation levels of queens and workers from GCD (genetic caste determination system) lineages were not significantly different until adulthood. Virgin queens had significantly higher relative levels of DNA methylation than workers (Smith et al., 2012). Therefore, the differences in methylation levels between queen and worker are widespread in social insects, although they vary with physiological state and developmental stage.

### The Key DMGs Involved in Reproduction, Morphology, and Vision Differentiation in Female Bees

Reproduction divergence is the most essential caste distinction between the queen and workers. The queen is fertile, while the worker is sterile; this difference is due to massive programmed cell death, which leads to the degeneration of 95–99% of the ovariole anlagen in workers during the final larval instar (Lago et al., 2016). We found 20 DMGs involved in reproduction that suggested the reproductive division of bees may be related to DNA methylation. A similar phenomenon exists in other animals; for example, DNA methylation is critical for high temperature induced Nile tilapia transsexualism (Wang et al., 2017). Ecdysteroid-regulated gene E74 is one of these. Ecdysteroid and ecdysteroid-regulated genes, including Broad-Complex (BR-C), E74 and E75, are involved in oogenesis in *Drosophila* and mosquito (Cho et al., 1995; Kozlova and Thummel, 2000; Sun et al., 2002). Paul et al. (2005) found that E74 expression in the adult queen abdomen was stronger than in the worker. Further, the localization of AmE74A transcripts in the ovariole varied by oogenesis stage, suggesting the involvement of AmE74A in oogenesis in the queen. Our GO annotation results showed that E74 regulated oogenesis. Although it is known that E74 is conserved in insect oocytogenesis, our current focus is on the role of E74 in programmed cell death. The crucial role of E74 has been demonstrated in the programmed cell death of *D. melanogaster* salivary glands (Jiang et al., 2000) and *B. mori* anterior silk glands (Sekimoto et al., 2007). Therefore, it was speculated that ecdysteroid-regulated gene E74 may be involved in regulating programmed cell death in the ovaries of worker bees. *Ash1* is involved in the regulation of histone methylation, which has been shown to regulate shoot regeneration in *Arabidopsis* by affecting the cell cycle (Liu et al., 2016). It was speculated that *ash1* gene may be involved in regulating cell cycle during the proto-oocyte division of honeybees, thereby affecting reproduction. RING finger protein 17 (*rfp17*) is an E3 ligase with a RING finger structure, which plays a role of recognizing target protein in protein ubiquitination. The RING finger protein family is widespread and functionally conserved in eukaryotes, from yeast to animals. The roles implicated in growth, oxidative stresses tolerance, response to the pathogen and signal transduction (Ardley, 2009; An et al., 2017; Zhang et al., 2019). *Rfp17* was annotated to be involved in gamete generation in our results. Although we found no evidence to prove that *rfp17* is directly related to gamete formation, the different methylation of *rfp17* between queen and worker larvae and its roles in other species suggested an important roles in castes differentiation. It is valuable to delve into this gene. Actin and actin-binding proteins have a key role in the transformation of fusomes to ring canals which connect the nurse cells to the oocyte in the newly formed follicles of the polytrophic meroistic *Drosophila* ovary (Robinson and Cooley, 1997; Spradling et al., 1997). *Protein hu-li tai shao*, encodes a homolog of adducing, is a gene required for ring canal formation during *Drosophila* oogenesis (Yue and Spradling, 1992). In our results, *Protein hu-li tai shao* was differentially methylated between queen and worker larvae at 3 and 5 days old, which possibly lead the interaction of actin and spectrin disorganized in worker larvae ovary.

*Dynamin*, which was differentially methylated between queen and worker, was annotated to be multifunctional. GO annotation showed that *dynamin* was involved in border/ovarian follicle cell migration, tissue/gland morphogenesis, and compound eye retinal cell programmed cell death. *Dynamin* was also shown to have versatile functions in *Drosophila*. *Dynamin*-mediated endocytosis is required for tube closure of the *Drosophila* ovary, prevention of photoreceptor degeneration, TGF-beta-superfamily homolog Dpp gradient formation, and synaptic vesicle recycling (Kruse et al., 2004; Rikhy et al., 2007; Pinal and Pichaud, 2011; Peters and Berg, 2016). Those annotated traits are all marker phenotypes distinguishing the queen and worker bees. Therefore, we speculate that this gene plays a key role in the differentiation of tissues and organs between queen bee and worker bee.

In addition to differences in external morphology, reproductive ability, and vision, other differences were found between the queen bee and the worker bee. Most remarkably, our results showed that DMGs associated with immunity and response to biotic and abiotic stress accounted for a significant proportion of the total DMGs between queen larvae and worker larvae (Supplementary Table S9). We hypothesized that there were significant differences in immune capacity and in the response to biotic and abiotic stress between queens and workers.

## Conclusion

The *A. mellifera* larval genome comprises approximately 49 million cytosines; of these, only about 90,000 cytosines were methylated. The methylated CpG account for 99% of the methylated cytosines. In *A. mellifera*, methylation mainly occurs in the exons. The methylation level of queen larvae was higher than that of worker larvae at 3 days old, but lower at 4 days old. At 5 days of age, the methylation levels of queen larvae and worker larvae were similar. The top 10 DMGs and 13 caste-specific methylated genes were speculated to correlate with caste determination. We also screened 38 DMGs between queen larvae and worker larvae involved in the differentiation of specific organs including those involved in reproduction, morphology, and vision differentiation. These genes are potential molecular markers for selective breeding of *A. mellifera* to improve fecundity, royal jelly production, and foraging, and represent candidate genes for investigation of specialized functional segregation during caste differentiation.

## Data Availability Statement

The datasets generated for this study can be found in the NCBI SRA accession: PRJNA640988.

## Author Contributions

HW, ZL, YW, LM, WZ, and BX participated in the conception and design of the study. HW drafted the manuscript and performed the experiments. HW and ZL analyzed the data. YW, LM, WZ, and BX revised the manuscript. All authors read and approved the final manuscript.

## Conflict of Interest

The authors declare that the research was conducted in the absence of any commercial or financial relationships that could be construed as a potential conflict of interest.

## Footnotes

1 http://www.genome.jp/kegg/

## Supplementary Material

FIGURE S1 The numbers of hyper-methylated genes and hypo-methylated genes worker vs. queen.

TABLE S1 Differentially Methylated Regions (DMRs) between queen larvae and worker larvae at the age of 3 days.

TABLE S2 Differentially Methylated Regions (DMRs) between queen larvae and worker larvae at the age of 4 days.

TABLE S3 Differentially Methylated Regions (DMRs) between queen larvae and worker larvae at the age of 5 days.

TABLE S4 GO annotation profile for differentially methylated genes (DMGs) between queen larvae and worker larvae at the age of 3 days.

TABLE S5 GO annotation profile for differentially methylated genes (DMGs) between queen larvae and worker larvae at the age of 4 days.

TABLE S6 GO annotation profile of differentially methylated genes (DMGs) between queen larvae and worker larvae at the age of 5 days.

TABLE S7 KEGG analysis of differentially methylated genes (DMGs).

TABLE S8 GO annotation and KEGG analysis of the top 10 DMGs between queen larvae and worker larvae at the age of 3, 4 and 5 days.

TABLE S9 The percent of DMGs involved in immunity and responses to biotic and abiotic stress.
