# Supplementary figures and images for: Genome-Wide Differential DNA Methylation in Reproductive, Morphological, and Visual System Differences Between Queen Bee and Worker Bee (Apis mellifera)
Source: Front Genet. 2020 Aug 7;11:770. doi: 10.3389/fgene.2020.00770 (PMC7438783; doi:10.3389/fgene.2020.00770)

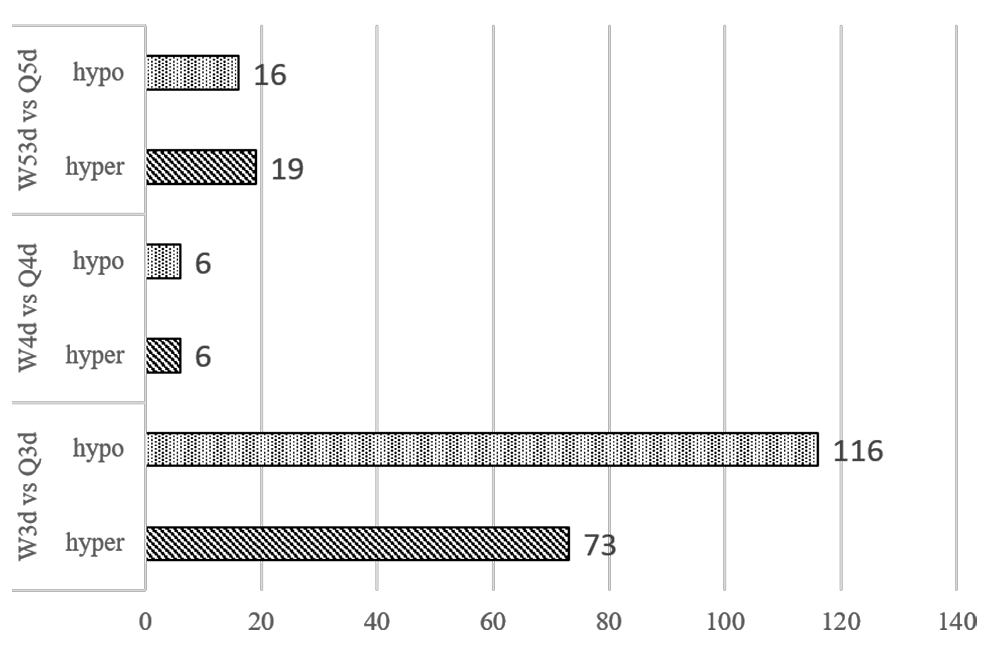

Supplement: FIGURE S1 — The numbers of hyper-methylated genes and hypo-methylated genes worker vs. queen. [file Image_1.TIF]
