# Supplementary material for: Genome-Wide Differential DNA Methylation in Reproductive, Morphological, and Visual System Differences Between Queen Bee and Worker Bee (Apis mellifera)
Source: Front Genet. 2020 Aug 7;11:770. doi: 10.3389/fgene.2020.00770 (PMC7438783; doi:10.3389/fgene.2020.00770)
Supplement: TABLE S6 — GO annotation profile of differentially methylated genes (DMGs) between queen larvae and worker larvae at the age of 5 days. [file Table_6.DOCX]

Table S9. DMGs involved in GO terms associated with immunity and response to biotic and abiotic stress.

| Days | Numers of GO terms | Numers of target GO terms | percent (%) | level | Average percent (%) |
| --- | --- | --- | --- | --- | --- |
| 3d | 320 | 23 | 7.19 | hyper | 6.27 |
|  | 543 | 29 | 5.34 | hypo |  |
| 4d | 282 | 12 | 4.26 | hyper | 6.27 |
|  | 435 | 36 | 8.28 | hypo |  |
| 5d | 345 | 13 | 3.77 | hyper | 3.23 |
|  | 186 | 5 | 2.69 | hypo |  |

Hyper: hyper methylation level worker larvae vs queen larvae. Hypo: hypo methylation level worker larvae vs queen larvae.
